# LAWS, REGULATIONS, AND POLICY: Genetically Engineered Salmon on the FDA’s Table

**DOI:** 10.1289/ehp.118-a384a

**Published:** 2010-09

**Authors:** David A. Taylor

**Affiliations:** **David A. Taylor** writes for *The Washington Post* and *Smithsonian* and is author of *Ginseng, the Divine Root*, about the science and subculture surrounding the medicinal plant. He teaches science writing at The Writer’s Center in Maryland

This fall the U.S. Food and Drug Administration (FDA) is expected to decide on the first-ever genetically engineered (GE) nonplant food source for human consumption—a proposal by Massachusetts-based AquaBounty to sell sterile eggs of a salmon with genetic material from the Chinook salmon and the ocean pout inserted for fast growth. Growers who buy the eggs can raise market-size salmon in 16–18 months instead of three years.^1^ But consumer groups have questioned what they call a secretive FDA process for evaluating foods developed from GE animals.

The rubric for regulating genetically modified animals differs from that used for crops, notes Greg Jaffe, biotechnology project director at the Center for Science in the Public Interest, a nonprofit consumer advocacy group in Washington, DC. “They use the animal-drug rubric because that’s the legal construct they have,” Jaffe says. “But it’s like jamming a square peg in a round hole” because there’s more secrecy in developing drugs than for foods. Still, he says, “it’s better than what we have on the crop side.”

Using the same legal basis as for animal drugs—and unlike the process for crops—the FDA’s process for animals is mandatory and requires approval before going to market. But although the FDA may provide a public-comment opportunity in conjunction with the Advisory Panel meeting it calls for each modified animal it reviews, Jaffe says the agency can issue its findings without providing all the underlying data, which requires consent from the company. “The FDA doesn’t control completely the transparency of its regulatory process,” Jaffe says; for that reason, he says, “I don’t think it goes far enough.”

The FDA process is outlined in a document called Guidance for Industry (GFI) 187,^2^ released in January 2009. According to Larisa Rudenko, senior adviser for biotechnology at the FDA Center for Veterinary Medicine, GFI 187 clarifies the agency’s statutory authority for regulating GE animals and lays out recommendations for how producers of those animals could submit data to the agency for review.

As described in GFI 187,^2^ the FDA requires a proposed GE change be stable for at least two noncontiguous generations sampled across a minimum span of three generations. The agency examines the health of the animal and the safety of any products from those animals that are consumed by humans, and assesses risk to the environment given the description of how the animal will be raised. The FDA uses a risk-based assessment of potential hazards and likelihood of harm.

Assessments are made on a case-by-case basis, emphasizes Rudenko. “We wanted to write a guidance at a sufficiently high altitude” that it would apply to all GE animals. She adds that “the FDA neither supports nor opposes [biotechnology]. We’re making a science-based decision.”

The divide over foods derived from GE animals has remained consistent for years, notes Marion Nestle, a professor in the Department of Nutrition, Food Studies, and Public Health at New York University. Compared with agricultural crops, she says, issues surrounding GE animals are mainly environmental, including concern that the animals could escape and breed with wild populations.

In the case of the AquaBounty AquAdvantage® salmon, only sterile female eggs will be sold to growers, and the fish will be grown in contained inland systems, according to the company’s application to the FDA. The main environmental concerns with the AquAdvantage salmon involve the possible impact on wild salmon, specifically the possibility that some eggs might not be sterile and end up being fertilized, and that the transgenic salmon might thus get established in marine ecosystems, where they could influence wild relatives, says Calestous Juma, director of the Science, Technology and Globalization Project at Harvard University.

“Though the chance of such events occurring remains minimal, it will be essential to monitor the technology’s use,” Juma says. Indeed, he adds, monitoring will address the main challenge of GE animals: how to generate trust in the new technology.

Given the depletion of fisheries stocks worldwide, says Juma, the AquAdvantage salmon could represent a significant way to increase protein production while reducing pressure on natural fish stocks. He calls it “one of the few examples where a new technology demonstrates clear human–environmental benefits,” with potential for improving food security globally. He adds that this technology could help developing nations bypass a growth stage of aquaculture involving heavy use of antibiotics and other chemicals, and instead leapfrog to more ecologically sound aquaculture.

“The public will see this has been a very thorough, careful evaluation,” says Rudenko. “We’re committed to making this [process] as transparent as we can.” She adds, “This is a mandatory approval process—what’s flexible is how one presents the data.”

As for what’s next for the AquAdvantage salmon, a representative for the company who requested to remain anonymous says AquaBounty received notification this summer that the agency had the bulk of the information needed for a decision.

## Figures and Tables

**Figure:**
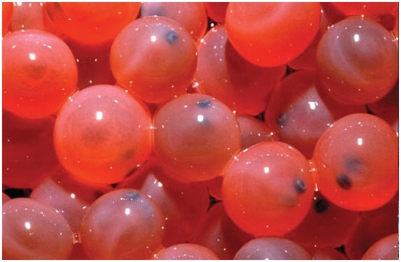


**Figure:**
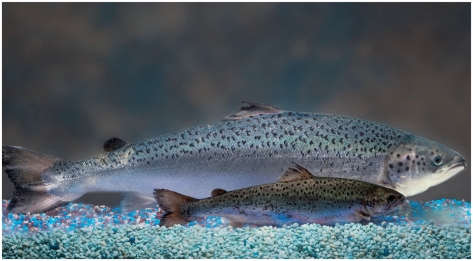
Sterile AquAdvantage^®^ eggs (opposite) will be sold to growers and yield only female fish (shown in rear, below, compared with a nontransgenic Atlantic salmon of the same age).
